# The Effect of Newly Designed High-Antioxidant Food Products on Oxidative Stress and Antioxidant Markers in Athletes

**DOI:** 10.3390/antiox14121457

**Published:** 2025-12-04

**Authors:** Kinga Zujko-Kowalska, Magdalena Stefanek, Izabela Łuszczewska, Łukasz Małek, Karol Adam Kamiński, Małgorzata Elżbieta Zujko

**Affiliations:** 1 Department of Cardiology and Internal Medicine with Cardiac Intensive Care Unit, M. Skłodowskiej-Curie 24A, 15-276 Białystok, Poland; kinga.zujko-kowalska@sd.umb.edu.pl (K.Z.-K.);; 2 Department of Population Medicine and Lifestyle Diseases Prevention, Medical University of Bialystok, Waszyngtona 15b, 15-269 Bialystok, Poland; 3 Student Scientific Society, Department of Food Biotechnology, Medical University of Białystok, Szpitalna 37, 15-295 Białystok, Poland; 4 Faculty of Rehabilitation, University of Physical Education, Marymoncka 34, 00-968 Warsaw, Poland; 5 Department of Food Biotechnology, Medical University of Białystok, Szpitalna 37, 15-295 Białystok, Poland

**Keywords:** diet, sport, antioxidants, polyphenols, healthy bars

## Abstract

The aim of this study was to develop functional food with high antioxidant potential and to examine its effect on oxidative–antioxidant markers in the blood of athletes in an intervention study. The study population consisted of 40 athletes—long-distance runners who were divided into a study group (SG) and a control group (CG). Before and after the dietary intervention in the blood, the following were determined: total antioxidant status (TAS), antioxidant enzymes (superoxide dismutase—SOD, glutathione peroxidase—GPx, catalase—CAT) and total oxidative stress (TOS). Additionally, the oxidative stress index (OSI) was calculated. It was shown that in the SG after the dietary intervention, the TOS (*p* < 0.001) and OSI (*p* = 0.029) decreased, while the TAS increased (*p* < 0.001). However, no significant differences were found in the level of antioxidant enzymes in the SG. In the CG, dietary intervention did not affect the level of the assessed parameters. This study demonstrated that functional food in the form of a bar with high antioxidant potential, rich in polyphenols, dietary fiber, vitamin E, selenium, and magnesium, can support the body’s endogenous antioxidant system and restore oxidative–antioxidant homeostasis in athletes. However, further studies are needed, including a larger group of athletes, longer intervention times, and different periods of the annual training cycle.

## 1. Introduction

Athletes are exposed to oxidative stress during training and competition. Intense physical activity causes increased oxygen consumption and, consequently, increased production of reactive oxygen species (ROS). An imbalance between antioxidants and oxidants leads to oxidative stress, which in athletes manifests itself as muscle fatigue and reduced physical performance. However, regular exercise can stimulate the body’s endogenous antioxidant system and post-training adaptation in athletes. Some authors have shown that the level of antioxidant markers is higher in groups of athletes compared to people with a sedentary lifestyle, which explains the role of physical activity in the prevention of chronic diseases [1]. If antioxidant defense is insufficient, oxidative damage to cells and tissues occurs. Athletes, especially men, are at risk of sudden death due to cardiovascular disease [2,3]. Therefore, during intense physical activity, it is important to ensure adequate rest after exercise and to follow an appropriate diet that will help restore the oxidative–antioxidant balance [4].

To improve physical performance, athletes often take antioxidant supplements. However, excessive consumption of dietary supplements can have harmful effects. High doses of antioxidant supplements hinder the beneficial post-training adaptations induced by ROS. Therefore, the greatest health benefits can be obtained by consuming natural antioxidants trough the diet. A well-planned diet supports the athlete’s training process, facilitates the body’s regeneration after exercise, and prevents the development of chronic diseases in this group. Dietary antioxidants stimulate the endogenous antioxidant defense system in athletes, which in turn may be crucial in preventing excessive oxidative damage and supporting regeneration [5].

Many studies in recent years have focused on the effects of exogenous antioxidant supplements, such as vitamins, minerals, and flavonoids, on oxidative stress and physical performance in athletes [6,7,8,9,10]. However, there are only a few studies assessing the effect of diet or functional foods on redox status in athletes [11,12,13]. The results obtained by other authors are not consistent, so we decided to check whether newly developed functional foods with high antioxidant potential can reduce oxidative stress in athletes.

The aim of this study was to develop a functional food in form of a bar with high antioxidant potential and to examine its effect on oxidative–antioxidant markers (total antioxidant status, antioxidant enzymes: superoxide dismutase, glutathione peroxidase, catalase, and oxidative stress) in the blood of athletes, long-distance runners, in an intervention study.

## 2. Materials and Methods

### 2.1. Prototype Design of New Antioxidant Bars

In the first stage of this study, commercially available functional foods for athletes were analyzed and 40 different products in the form of bars were purchased in triplicate to determine their polyphenol content and antioxidant potential. We tested bars labeled “healthy bars”, “healthy calorie bars”, and “healthy bars for athletes”. All purchased bars were stored at room temperature before laboratory analysis. Total polyphenol content (TPC) in food was determined using Folin–Ciocalteau reagent and expressed as gallic acid equivalents (mg GAE/100 g) [14], and total antioxidant potential was determined by the FRAP (ferric ion reducing antioxidant potential) method and expressed as mmol Fe^2+^/100 g [15]. It was found that the mean TPC in analyzed bars was 74.3 ± 32.5 mg/100 g (range: 58.6–100.0 mg/100 g), and the mean FRAP was 1.8 ± 1.1 mmol/100 g (range: 1.2–2.9 mmol/100 g).

Of all the bars purchased in the store, we selected the one with the highest antioxidant potential (FRAP—2.9 mmol/100 g) and the highest polyphenol content (TPC—100 mg/100 g), and assigned it to the control group. This bar contained the following ingredients: peanuts, dates, honey, raisins, rice. The bar packaging provides its basic nutritional value per 100 g: energy—526 kcal, fat—32 g, carbohydrates—40 g, protein—16 g, fiber—5.1 g.

In the next stage, the antioxidant properties of food were analyzed based on our previously published databases [16] and those available online [17,18]. In these databases, the antioxidant potential of food products was determined using the FRAP method. On this basis, foods with the highest FRAP were selected and used to develop prototypes of 3 bars with high antioxidant potential. The design of new bars and the production of bar prototypes took place at the Biotechnology Laboratory (Białystok, Poland).

Bar no. 1 consisted of nuts (peanuts, almonds), sunflower seeds, fruit (dates, cranberries), and buckwheat honey. Bar no. 2 consisted of nuts (cashews, Brazil nuts, hazelnuts, peanuts, pecans, pistachios, almonds, coconut), sunflower seeds, fruits (dates, cherries, cranberries), and rapeseed honey. And bar no. 3 was made with fruit (mango, dates, pineapple, goji, cranberries, chokeberries), chia seeds, raspberry juice, and rapeseed honey.

The nutritional value and acceptability of the products by potential consumers were assessed in an accredited Hamilton Laboratory (Gdynia, Poland). For the consumer research, 60 individuals were randomly selected from the group of interested individuals who met the inclusion criteria established for the study group in the intervention study (men, 20–30 years old, training at least 4 times a week for a minimum of 90 min, with no chronic diseases, non-smokers, and with no dietary supplements). Before participating in the consumer study, individuals completed a study qualification questionnaire. In the consumer survey, a hedonic scale from 1 to 8 was used (where 1 means “I really dislike” and 8 means “I really like”), and the intention to purchase the product was assessed on a scale from 1 to 5 (where 1 means low and 5 means high purchase intention).

Additionally, TPC [14] and FRAP [15] were determined in the designed bars at the Biotechnology Laboratory (Białystok, Poland).

### 2.2. Study Population and Intervention

This interventional study was registered as a clinical trial (SportDiet, no.: NCT06855979, date: 25 February 2025) and approved by the Ethics Committee of the Medical University of Białystok (no.: APK.002.405.2024, date: 24 October 2024).

The study population consisted of 40 men—long-distance runners aged 20–30 years. People participating in the study were recruited from sports clubs and sports associations in Białystok city. Inclusion criteria for the study included age (20–30 years), gender (men), training at least 4 times a week (90–120 min per session), no chronic diseases, no smoking, and no dietary supplements for 1 month before and during the intervention. All study participants agreed to follow the principles of the interventional study. Athletes’ adherence to the study guidelines was assessed in a follow-up survey conducted after the study concluded.

People who qualified for this study were divided into two groups: the study group (SG, n = 20) and the control group (CG, n = 20). The intervention study lasted 1 month and was conducted during the preparatory period before the competition. The SG received a daily bar designed specifically for this study, while the CG received 1 bar purchased from a store. For the CG we selected the bar with the highest antioxidant potential and polyphenol content among commercially available bars. Finally, the antioxidant potential of the SG bar was 4 times higher and the polyphenol content 6 times higher (FRAP—5.85 mmol/50 g, TPC—315 mg/50 g) than the CG bar (FRAP—1.45 mmol/50 g, TPC—50 mg/50 g).

Basic data about the study population (e.g., age, weight, height, education level) were collected using self-administered questionnaires.

Dietary information before and after the intervention period was collected using 3-day food diaries (2 working days and 1 weekend day). Dietary energy and nutrient intakes were calculated using the Diet 6.0 computer program (National Institute of Public Health, Warsaw, Poland). Dietary antioxidant potential and dietary polyphenol intake were determined by multiplying the average daily intake of individual food products by the antioxidant potential and polyphenol content of these products, as described in a previous study [16], based on available databases [17,18].

### 2.3. Antioxidant and Oxidative Stress Markers

Blood was obtained before and after the intervention period to measure antioxidant status, antioxidant enzymes (superoxide dismutase, glutathione peroxidase, catalase) and oxidative stress. The determinations were performed by the spectrophotometric method using a microplate reader (Infinite M200 Pro Tecan, Männedorf, Switzerland). The oxidative stress index (OSI) was calculated as the ratio of total oxidant status (TOS) to total antioxidant status (TAS). Blood samples were collected in the morning (7–8 AM), following the overnight (12 h) fast, into tubes containing EDTA as an anticoagulant. Athletes were advised to refrain from exercise for 48 hr before blood sampling. Samples were centrifuged at 3000× *g* for 10 min at 4 °C, and the top yellow plasma layer was then pipetted off. Plasma samples were stored in a freezer at −80 °C for no longer than one month before analysis, according to the kit manufacturers’ recommendations.

Total antioxidant status was determined using the ImAnOx (TAS/TAC) kit (Immundiagnostik AG, Bensheim, Germany). The principle part of the method is the reaction of antioxidants contained in the sample with a specific amount of exogenously provided hydrogen peroxide. The antioxidants in the sample eliminate a certain amount of the provided hydrogen peroxide. The residual hydrogen peroxide was determined photometrically by an enzymatic reaction and measured at 450 nm. The mean intra-assay coefficient of variation (calculated for ten duplicate samples) was 3.2%.

Total oxidative status was performed using the PerOx (TOS/TOC) Kit (Immundiagnostik AG, Bensheim, Germany). In this method, the determination of peroxides is performed by the reaction of a peroxidase with peroxides in the sample. Samples were measured at 450 nm. The mean intra-assay coefficient of variation (calculated for ten duplicate samples) was 2.9%.

Catalase (CAT) is an antioxidant enzyme involved in the conversion of hydrogen peroxide to oxygen and water. Catalase Assay Kit (No. 707002, Cayman Chemical Company, Ann Arbor, MI, USA) is based on the reaction of the enzyme with methanol in the presence of an optimal concentration of hydrogen peroxide. The formaldehyde produced is measured colorimetrically with 4-amino-3-hydrazino-5-mercapto-1,2,4-triazole (Purpald) as the chromogen. Purpald specifically forms a bicyclic heterocycle with aldehydes, which upon oxidation changes from colorless to a purple color. Samples were measured at 540 nm. The mean intra-assay coefficient of variation (calculated for ten duplicate samples) was 3.8%.

Superoxide Dismutases (SODs) are metalloenzymes that catalyze the dismutation of the superoxide anion to molecular oxygen and hydrogen peroxide, and thus form a crucial part of the cellular antioxidant defense system. The Superoxide Dismutase Assay Kit (No. 706002, Cayman Chemical Company, Ann Arbor, MI, USA) utilizes a tetrazolium salt for the detection of superoxide radicals generated by xanthine oxidase and hypoxanthine. One unit of SOD is defined as the amount of enzyme needed to exhibit 50% dismutation of the superoxide radical measured in change in absorbance per minute at 25 °C and pH = 8. Samples were measured at 450 nm. The mean intra-assay coefficient of variation (calculated for ten duplicate samples) was 3.2%.

Glutathione peroxidase (GPx) catalyzes the reduction of hydroperoxides, including hydrogen peroxide, by reduced glutathione, and functions to protect the cells from oxidative damage. The glutathione peroxidase Assay Kit (No. 703102, Cayman Chemical Company, Ann Arbor, MI, USA) measures GPx activity indirectly by a coupled reaction with glutathione reductase (GR). Oxidized glutathione (GSSG), produced upon reduction of hydroperoxide by GPx, is recycled to its reduced state by GR and NADPH. The oxidation of NADPH to NADP+ is accompanied by a decrease in absorbance at 340 nm. Under conditions in which the GPx activity is rate-limiting, the rate of decrease in absorbance is directly proportional to the GPx activity in the sample. Samples were measured at 340 nm. The mean intra-assay coefficient of variation (calculated for ten duplicate samples) was 5.7%.

### 2.4. Statistical Analysis

Statistical analysis of the data was performed using the Statistica program (version 14.1.0.4 PL; TIBCO Software Inc., Palo Alto, CA, USA). Continious variables were presented as the mean and standard deviation (M ± SD) or median and interquartile range (Me, IQR), whereas categorical variables were presented as number and percentage (N, %). Continuous variables were compared using Student’s *t*-test or Mann–Whitney U test, depending on the results of the test for normality of the distribution. Normality of continuous data distribution was verified with the Shapiro–Wilk test. The Anova test was used to compare the consumer acceptability of the 3 new bars. The Mann–Whitney U test was used to compare antioxidant and oxidative markers before and after the intervention. Categorical variables were assessed using the chi-square test. In all statistical tests, *p* < 0.05 was considered statistically significant.

## 3. Results

### 3.1. Nutritional and Antioxidant Value of the Bars

The nutritional and antioxidant values of the designed bars are presented in Table 1. Bar no. 2 was characterized by the highest antioxidant potential, and the highest content of polyphenols, selenium, magnesium, zinc, MUFA, PUFA, and vitamin E.

### 3.2. Consumer Acceptability of the Bars

The consumer acceptability of new bars is presented in Table 2.

It was shown that taste rating, overall product rating, and purchase intention were significantly higher for Bar no. 1 and Bar no. 2 compared to Bar no. 3.

Based on the nutritional and antioxidant value assessment and consumer opinions, Bar no. 2 was selected for intervention study. The average weight of all bars was approximately 50 g.

### 3.3. Study Population and Intervention Study

The main characteristics of the study population are summarized in Table 3. The study and control groups were matched for age, gender, BMI, and education level. The mean age was 24.4 ± 5.3, and the mean BMI was 23.1 ± 1.9. Over 82% of participants had a higher education.

The nutritional and antioxidant value of the diet before and after the intervention period is presented in Table 4. It was shown that the nutritional and antioxidant value of the athletes’ diet did not differ significantly before and after the dietary intervention period. It is worth noting that the average intake of individual nutrients was in line with the recommended daily intake for non-athletes in the Polish population, with the exception of vitamin D. The average intake of vitamin D was 3 times lower than the recommended intake [19]. However, there are no standards specifying the daily intake of polyphenols and the value of the antioxidant potential of the diet.

**Figure 1:**
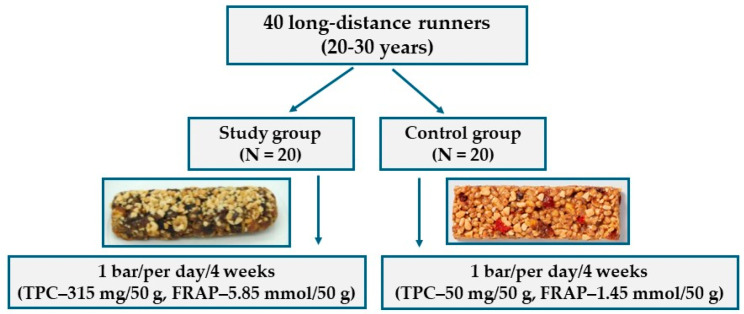
Participant flowchart.

A flowchart of the study population is presented in Figure 1. The research group received a bar with an antioxidant potential four times higher than the control group bar. According to the athletes’ declarations, all participants in the study and control groups met the study conditions and consumed one provided antioxidant bar per day.

### 3.4. Oxidative–Antioxidant Markers Before and After Intervention

**Figure 2:**
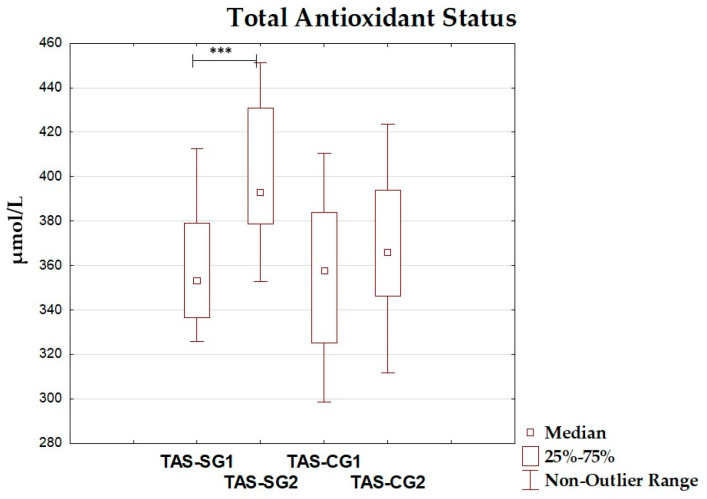
Median and interquartile range of total antioxidant status before and after the dietary intervention; ***—*p* < 0.001; TAS-SG1—total antioxidant status before intervention in the study group; TAS-SG2—total antioxidant status after intervention in study group; TAS-CG1—total antioxidant status before intervention in the control group; TAS-CG2—total antioxidant status after intervention in the control group.

**Figure 3:**
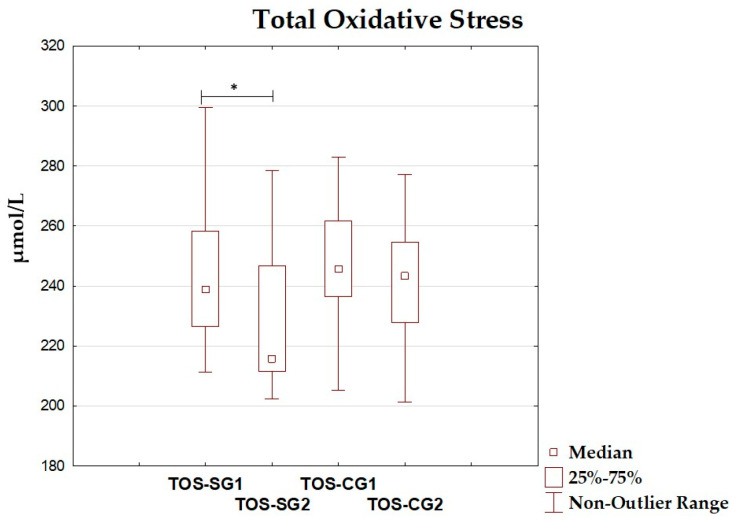
Median and interquartile range of total oxidative stress before and after the dietary intervention; *—*p* < 0.05; TOS-SG1—total oxidative status before intervention in the study group; TOS-SG2—total oxidative status after intervention in the study group; TOS-CG1—total oxidative status before intervention in the control group; TOS-CG2—total oxidative status after intervention in the control group.

**Figure 4:**
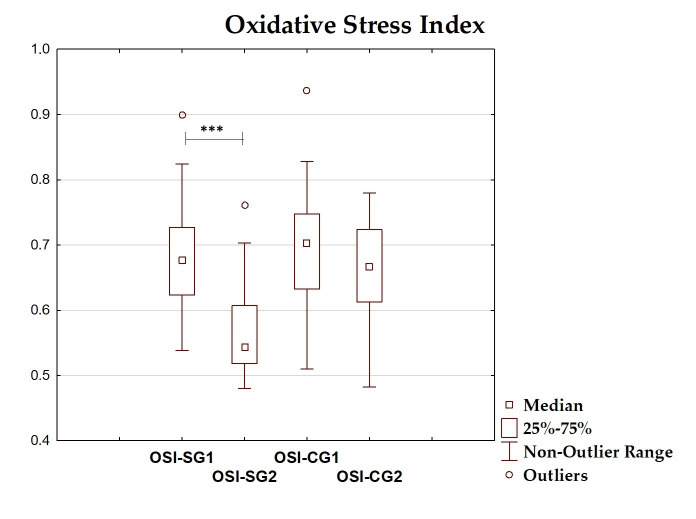
Median and interquartile range of oxidative stress index before and after the dietary intervention; ***—*p* < 0.001; OSI-SG1—oxidative stress index before intervention in the study group; OSI-SG2—oxidative stress index after intervention in the study group; OSI-CG1—oxidative stress index before intervention in the control group; OSI-CG2—oxidative stress index after intervention in the control group.

**Figure 5:**
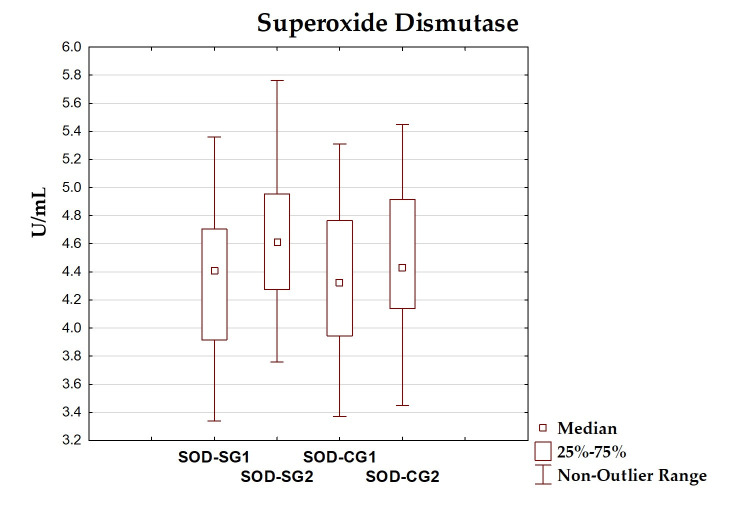
Median and interquartile range of superoxide dismutase before and after the dietary intervention; SOD-SG1—superoxide dismutase before intervention in the study group; SOD-SG2—superoxide dismutase after intervention in the study group; SOD-CG1—superoxide dismutase before intervention in the control group; SOD-CG2—superoxide dismutase after intervention in the control group.

**Figure 6:**
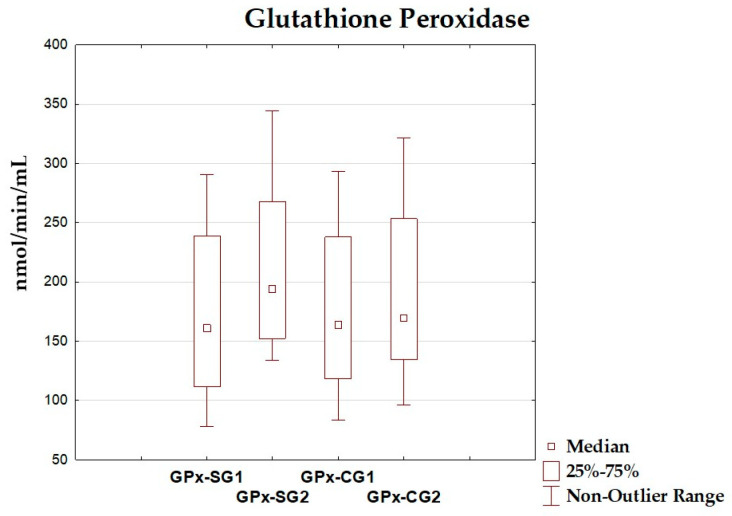
Median and interquartile range of glutathione peroxidase before and after the dietary intervention; GPx-SG1—glutathione peroxidase before intervention in the study group; GPx-SG2—glutathione peroxidase after intervention in the study group; GPx-CG1—glutathione peroxidase before intervention in the control group; GPx-CG2—glutathione peroxidase after intervention in the control group.

**Figure 7:**
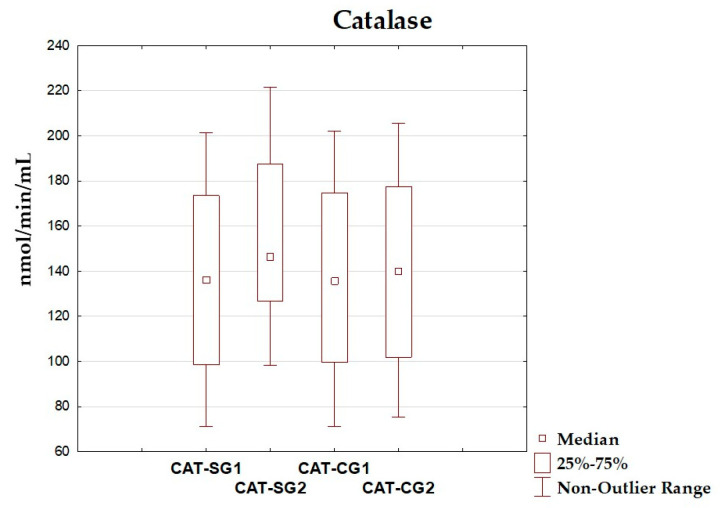
Median and interquartile range of catalase before and after the dietary intervention; CAT-SG1—catalase before intervention in the study group; CAT-SG2—catalase after intervention in the study group; CAT-CG1—catalase before intervention in the control group; CAT-CG2—catalase after intervention in the control group.

The differences between total antioxidant status, total oxidant status, oxidative stress index, and antioxidant enzyme (superoxide dismutase, glutathione peroxidase, catalase) levels before and after the dietary intervention period are presented in Table 5 and Figure 2, Figure 3, Figure 4, Figure 5, Figure 6 and Figure 7. It was shown that in the SG, after the dietary intervention, the total oxidative status (*p* < 0.001) and oxidative stress index (*p* = 0.029) decreased, while the level of total antioxidant status increased (*p* < 0.001). However, no significant differences were found in the level of antioxidant enzymes in the SG. In the CG, dietary intervention did not affect the level of the assessed parameters.

## 4. Discussion

A well-balanced and diversified diet, rich in natural antioxidants such as polyphenols, vitamin E, C, and carotenoids, as well as minerals (Se, Mn, Zn, Cu, Fe), which are part of endogenous antioxidant enzymes, is essential for the proper functioning of the body, especially during intense physical exercise [20,21]. The results of this intervention study showed that consuming foods rich in natural antioxidants has a beneficial effect on increasing total antioxidant status and reducing oxidative stress. An increase in antioxidant enzyme activity was also observed after the intervention period, but these values were not statistically significant. It is worth noting that the nutritional value of the athletes’ diet did not differ before and after the intervention period. This study involved a homogeneous group of young athletes, long-distance runners, and all participants in the SG and CG met the study conditions and consumed one provided antioxidant bar per day. A food product in the form of a bar was selected for the intervention studies, which is an easily accessible form of food after training. A newly designed bar for the research group contained the following ingredients: nuts (cashews, Brazil nuts, hazelnuts, peanuts, pecans, pistachios, almonds, coconut), sunflower seeds, fruits (dates, cherries, cranberries), and rapeseed honey. This bar had the highest antioxidant potential and polyphenol content of all our newly designed bars. In addition, we showed that our bar was a source of dietary fiber, magnesium, selenium, and vitamin E. All these nutrients contributed to the high antioxidant potential of the bar. According to Regulation (EC) no. 1924/2006 of the European Parliament and of the Council of 20 December 2006 on nutrition and health claims made on foods [22], a product is a source of fiber if it contains at least 3 g of fiber per 100 g, and is a source of vitamins and minerals if a portion of the product contains at least 15% of the reference daily intake. There are no legal regulations or consumption recommendations regarding polyphenols or the antioxidant potential of diet. However, observational studies have shown that a diet with high antioxidant potential and polyphenol content has a beneficial effect on health [23,24]. Similarly, experimental studies indicated that a plant extract rich in polyphenols can protect against oxidative stress in in vitro and in vivo mouse models [25,26]. Dietary polyphenols, including phenolic acids, flavonoids, lignans, and stilbenes, are bioactive compounds commonly found in plant foods, such as fruits, vegetables, and nuts. Polyphenols exhibit antioxidant properties due to their chemical structure. They have hydroxyl groups (-OH) attached to aromatic rings. These groups enable the neutralization of free radicals by donating electrons or hydrogen atoms, thus protecting cells from damage [27]. Polyphenols and dietary fiber work synergistically. Dietary fiber nourishes the gut microbiome, which metabolizes polyphenols into bioactive compounds, supporting the body’s metabolic and immune homeostasis [28]. Vitamin E is a major lipid-soluble antioxidant that effectively scavenges peroxyl radicals in biological membranes and low-density lipoproteins [29]. Selenium is an essential component of selenoproteins, including the antioxidant enzyme glutathione peroxidase [30]. Selenium is a trace element deficient in the diet of Polish residents, primarily due to the low content of this element in Polish soils. Therefore, even a properly planned diet may not contain sufficient selenium [31]. Magnesium supports the action of antioxidant enzymes (catalase, superoxide dismutase), and it was shown that a diet low in magnesium leads to elevated levels of oxidative markers [32].

This study demonstrated the benefits of consuming foods with high antioxidant potential, which are sources of polyphenols, dietary fiber, selenium, magnesium, and vitamin E. The results of these studies are in some respects consistent with reports by other authors. Schneider et al. [11] showed that a 2-week antioxidant diet improved antioxidant capacity and SOD activity in triathletes, but no changes in GPx activity were observed. Gaamouri et al. [33] demonstrated that supplementation with carob-rich polyphenols during a 6-week training program reduced plasma lipid peroxidation MDA (malondialdehyde) and improved CAT and SOD activity, as well as physical performance, in taekwondo athletes. McAnulty et al. [13] found that a 7-day supplementation of 150 g of blueberries per day resulted in a reduction in lipid peroxidation markers in athletes training in a hot environment, but did not show such an effect with vitamin C supplementation at a dose of 1250 mg per day. Kawamura et al. [34] showed that 200 mg of oligomerized polyphenols per day contained in lychee fruit extract can improve the physical performance of male athletes during high-intensity exercise. However, Valder et al. did not observe a significant effect of short-term (6-day) use of red fruit juice (25% chokeberry) on oxidative stress and inflammatory processes during an intense endurance exercise program in athletes. The authors explained that the lack of effect was due to the short duration of the intervention and too low a content of polyphenols in the product intended for intervention [35]. Better results can be achieved by systematically consuming foods containing antioxidants. Bianchi et al. demonstrated the positive effect of a Mediterranean diet rich in natural antioxidants on the increase in total plasma antioxidant activity and the reduction in markers of oxidative stress and inflammation in athletes [36]. Adhering to specific dietary patterns is difficult. Therefore, we decided to design a product in an easily accessible form of a bar that can supplement a diet with natural antioxidants.

In our study, no statistically significant differences were found between the antioxidant enzymes SOD and GPx and CAT activity before and after the intervention period during preparation for competition. The activity of antioxidant enzymes may change depending on the period of the training macrocycle. Sadowska-Krępa et al. [37] showed the highest activity of SOD and CAT in the competition period in comparison to the preparation and transition periods, and GPx in the transition period in comparison to the preparatory and competition period in American football players and soccer players.

This study has some strengths and limitations. The strength of this study is that all participants completed the intervention period without dropping out and met the study conditions. Next, the study group was homogeneous, matched in terms of age, gender, smoking status, BMI, and education. Additionally, the intervention study used a high-antioxidant bar specifically designed for this study. In the bar intended for the study group, we determined the total antioxidant potential, total polyphenol content, and nutritional value, including the content of antioxidant vitamins and minerals. Finally, the participants’ diets did not differ in terms of nutritional and antioxidant value before and after the intervention, allowing for an assessment of the effects of the designed bar on antioxidant status, oxidative stress, and antioxidant enzyme activity. This study was limited by its small sample size and short study period. Further studies are needed, including a larger group of athletes, longer intervention times, and different periods of the annual training cycle. Research can also be extended to determine polyphenol profiles and examine the impact of dietary antioxidants on the physical performance of athletes.

## 5. Conclusions

Foods with high antioxidant potential, rich in polyphenols, dietary fiber, vitamin E, selenium, and magnesium, may support the body’s endogenous antioxidant system and restore oxidant–antioxidant homeostasis. However, further interventional studies are needed to confirm these results. The designed bar was tested in an interventional study involving a group of athletes, but it may also be useful for other population groups in the prevention of chronic diseases related to oxidative stress.

## Figures and Tables

**Table 1:** Nutritional and antioxidant value of the designed bars.

Variables	Units	Bar No. 1	Bar No. 2	Bar No. 3
Energy	kcal/100 g	434	458	308
Protein	g/100 g	10.4	12.0	2.9
Carbohydrates	g/100 g	47.2	40.2	69.6
Dietary fiber	g/100 g	9.9	7.8	6.1
Fat	g/100 g	20.4	28.4	0.6
SFA	g/100 g	1.8	4.3	0.2
MUFA	g/100 g	11.7	16.3	0.2
PUFA	g/100 g	6.8	7.8	0.2
Zinc	mg/100 g	1.5	1.9	0.5
Phosphorus	mg/100 g	218	257	49.5
Magnesium	mg/100 g	120	135	51.8
Potassium	mg/100 g	521	544	635
Selenium	µg/100 g	7.43	22.3	2.5
Calcium	mg/100 g	114	99.1	55.3
Iron	mg/100 g	1.7	2.3	1.9
Vitamin B1	mg/100 g	0.12	0.2	0.08
Vitamin B2	mg/100 g	0.27	0.16	0.1
Vitamin B3	mg/100 g	1.9	1.7	0.7
Vitamin B9	µg/100 g	14.7	10.4	9.4
Vitamin E	mg/100 g	5.4	8.4	1.0
TPC	mg GAE/100 g	546	630	385
FRAP	mmol/100 g	8.4	11.7	5.2

SFA—saturated fatty acids, MUFA—monounsaturated fatty acids, PUFA—polyunsaturated fatty acids, TPC—total polyphenol content, FRAP—ferric ion reducing antioxidant potential.

**Table 2:** Consumer acceptability of new antioxidant bars.

Scale	Range	Bar No. 1	Bar No. 2	Bar No. 3	*p*
Hedonic scale	1–8				
Taste		7.37 ± 0.53	7.30 ± 0.64	7.05 ± 0.84	0.045 _(1,2/3)_
Overall rating		7.32 ± 0.47	7.32 ± 0.59	6.97 ± 0.76	0.039 _(1,2/3)_
Intention to purchase	1–5	4.62 ± 0.35	4.55 ± 0.41	4.20 ± 0.74	0.027 _(1,2/3)_

1—bar No. 1, 2—bar No. 2, 3—bar No. 3.

**Table 3:** Basic characteristics of the study population.

Characteristics	Total	Study Group	Control Group	*p*
Sample number	40	20	20	-
Age, years	24.4 ± 5.3	24.8 ± 4.7	23.9 ± 5.6	0.723
Gender (men), N (%)	100	100	100	-
Higher education, N (%)	82.5	85	80	0.644
BMI (kg/m^2^)	23.1 ± 1.9	23.7 ± 1.6	22.9 ± 2.3	0.572

N—number, BMI—body mass index.

**Table 4:** Nutritional and antioxidant value of the diet before and after intervention.

Variables	SG Before	SG After	*p*	CG Before	CG After	*p*
Energy (kcal)	2465 ± 414	2512 ± 494	0.341	2410 ± 562	2548 ± 470	0.561
Protein (g)	92.6 ± 12.7	90.4 ± 17.3	0.423	88.9 ± 19.5	90.3 ± 15.2	0.385
Carbohydrates (g)	324.3 ± 45.5	343.4 ± 54.2	0.092	295.7 ± 35.2	320.3 ± 42.1	0.113
Dietary fiber (g)	24.3 ± 8.4	23.8 ± 9.2	0.655	24.5 ± 7.2	24.1 ± 8.6	0.726
Fat (g)	83.4 ± 24.2	78.3 ± 26.6	0.543	85.7 ± 22.9	82.9 ± 25.3	0.584
SFA (g)	24.4 ± 11.5	23.8 ± 12.1	0.241	23.5 ± 12.7	24.1 ± 12.5	0.183
MUFA (g)	31.4 ± 11.7	32.1 ± 12.5	0.523	29.5 ± 12.9	32.8 ± 13.7	0.127
PUFA (g)	13.2 ± 5.8	12.8 ± 4.7	0.146	13.5 ± 6.1	13.7 ± 6.8	0.454
Magnesium (mg)	465.3 ± 88.2	446.8 ± 85.3	0.352	446.2 ± 98.7	428.1 ± 91.5	0.148
Zinc (mg)	11.3 ± 4.8	10.8 ± 4.2	0.395	11.5 ± 3.9	10.4 ± 3.7	0.092
Iron (mg)	12.2 ± 6.8	13.2 ± 7.6	0.144	12.6 ± 7.1	13.5 ± 8.2	0.122
Vitamin C (mg)	105.2 ± 63.7	101.9 ± 70.4	0.321	103.3 ± 75.8	106.2 ± 77.6	0.246
Vitamin E (mg)	11.4 ± 5.6	12.1 ± 6.3	0.286	11.8 ± 6.7	12.7 ± 7.4	0.183
Vitamin A (mg)	1125 ± 487	1231 ± 423	0.164	1096 ± 459	1198 ± 511	0.086
Vitamin D (µg)	5.3 ± 3.4	5.8 ± 4.1	0.113	5.1 ± 3.9	5.5 ± 4.4	0.144
TPC (mg)	2311 ± 643	2287 ± 711	0.321	2365 ± 574	2295 ± 624	0.286
FRAP (mmol)	15.3 ± 7.1	14.6 ± 7.5	0.487	15.8 ± 6.9	14.9 ± 6.7	0.382

Variables are presented as mean and standard deviation (M ± SD), SFA—saturated fatty acids, MUFA—monounsaturated fatty acids, PUFA—polyunsaturated fatty acids, TPC—total polyphenol content, FRAP—ferric ion reducing antioxidant potential.

**Table 5:** Oxidative–antioxidant markers before and after the intervention period.

Variables	Study Group Before Intervention		Study Group After Intervention		*p*
	M ± SD	Me (IQR)	M ± SD	Me (IQR)	
TAS (µmol/L)	359.60 ± 28.21	353.21 (336.43–378.97)	401.98 ± 32.21	392.82 (378.83–430.78)	<0.001
TOS (µmol/L)	244.72 ± 25.21	238.93 (226.47–258.29)	227.54 ± 22.87	215.55 (211.46–246.77)	0.029
OSI	0.686 ± 0.089	0.677 (0.623–0.727)	0.569 ± 0.076	0.544 (0.519–0.607)	<0.001
SOD (U/mL)	4.318 ± 0.572	4.410 (3.915–4.705)	4.646 ± 0.559	4.610 (4.275–4.955)	0.075
GPx (nmol/min/mL)	171.73 ± 71.29	161.15 (111.54–238.99)	210.65 ± 66.47	194.60 (152.37–267.60)	0.082
CAT (nmol/min/mL)	135.06 ± 41.85	135.98 (98.58–173.43)	157.15 ± 37.85	146.54 (126.57–187.51)	0.088
**Variables**	**Control Group Before Intervention**		**Control Group After Intervention**		***p***
	**M ± SD**	**Me (IQR)**	**M ± SD**	**Me (IQR)**	
TAS (µmol/L)	355.58 ± 35.48	357.74 (325.28–383.82)	368.21 ± 34.49	365.92 (346.29–393.97)	0.261
TOS (µmol/L)	246.26 ± 21.67	245.70 (236.46–261.74)	240.49 ± 21.53	243.43 (227.82–254.60)	0.404
OSI	0.699 ± 0.095	0.703 (0.633–0.748)	0.658 ± 0.080	0.667 (0.613–0.724)	0.403
SOD (U/mL)	4.309 ± 0.555	4.320 (3.945–4.765)	4.446 ± 0.545	4.430 (4.140–4.915)	0.434
GPx (nmol/min/mL)	173.28 ± 70.46	163.65 (118.16–237.77)	185.92 ± 72.54	170.02 (134.49–253.28)	0.579
CAT (nmol/min/mL)	135.68 ± 42.08	135.78 (99.53–174.79)	140.16 ± 41.87	139.98 (101.87–177.49)	0.738

M—mean, SD—standard deviation, Me—median, IQR—interquartile range, TAS—total antioxidant status, TOS—total oxidant status, OSI—oxidative stress index, SOD—superoxide dismutase, GPx—glutathione peroxidase, CAT—catalase.

## Data Availability

The original contributions presented in this study are included in the article. Further inquiries can be directed to the corresponding author.
